# Removal of glucuronic acid from xylan is a strategy to improve the conversion of plant biomass to sugars for bioenergy

**DOI:** 10.1186/s13068-017-0902-1

**Published:** 2017-09-19

**Authors:** Jan J. Lyczakowski, Krzysztof B. Wicher, Oliver M. Terrett, Nuno Faria-Blanc, Xiaolan Yu, David Brown, Kristian B. R. M. Krogh, Paul Dupree, Marta Busse-Wicher

**Affiliations:** 1 Department of Biochemistry, University of Cambridge, Tennis Court Road, Cambridge, CB2 1QW UK; 2 The Gurdon Institute, University of Cambridge, Tennis Court Road, Cambridge, CB2 1QN UK; 3 Natural Material Innovation Centre, University of Cambridge, 1 Scroope Terrace, Cambridge, CB2 1PX UK; 4 OpenPlant Synthetic Biology Research Centre, Department of Plant Sciences, University of Cambridge, Downing Site, Cambridge, CB2 3EA UK; 5 Department of Protein Biochemistry and Stability, Novozymes A/S, Krogshøjvej 36, 2880 Bagsværd, Denmark; 6 Ossianix, Stevenage Bioscience Catalyst, Gunnels Wood Rd, Stevenage, Hertfordshire, SG1 2FX UK; 7 Present Address: Shell Global Solutions International BV, Lange Kleiweg 40, 2288 GK Rijswijk, The Netherlands

**Keywords:** Biofuels, Xylan, Glucuronic acid, Conifers, Softwood, GUX

## Abstract

**Background:**

Plant lignocellulosic biomass can be a source of fermentable sugars for the production of second generation biofuels and biochemicals. The recalcitrance of this plant material is one of the major obstacles in its conversion into sugars. Biomass is primarily composed of secondary cell walls, which is made of cellulose, hemicelluloses and lignin. Xylan, a hemicellulose, binds to the cellulose microfibril and is hypothesised to form an interface between lignin and cellulose. Both softwood and hardwood xylan carry glucuronic acid side branches. As xylan branching may be important for biomass recalcitrance and softwood is an abundant, non-food competing, source of biomass it is important to investigate how conifer xylan is synthesised.

**Results:**

Here, we show using Arabidopsis *gux* mutant biomass that removal of glucuronosyl substitutions of xylan can allow 30% more glucose and over 700% more xylose to be released during saccharification. Ethanol yields obtained through enzymatic saccharification and fermentation of *gux* biomass were double those obtained for non-mutant material. Our analysis of additional xylan branching mutants demonstrates that absence of GlcA is unique in conferring the reduced recalcitrance phenotype. As in hardwoods, conifer xylan is branched with GlcA. We use transcriptomic analysis to identify conifer enzymes that might be responsible for addition of GlcA branches onto xylan in industrially important softwood. Using a combination of in vitro and in vivo activity assays, we demonstrate that a white spruce (*Picea glauca*) gene, *PgGUX*, encodes an active glucuronosyl transferase. Glucuronic acid introduced by PgGUX reduces the sugar release of Arabidopsis *gux* mutant biomass to wild-type levels indicating that it can fulfil the same biological function as native glucuronosylation.

**Conclusion:**

Removal of glucuronic acid from xylan results in the largest increase in release of fermentable sugars from Arabidopsis plants that grow to the wild-type size. Additionally, plant material used in this work did not undergo any chemical pretreatment, and thus increased monosaccharide release from *gux* biomass can be achieved without the use of environmentally hazardous chemical pretreatment procedures. Therefore, the identification of a gymnosperm enzyme, likely to be responsible for softwood xylan glucuronosylation, provides a mutagenesis target for genetically improved forestry trees.

**Electronic supplementary material:**

The online version of this article (doi:10.1186/s13068-017-0902-1) contains supplementary material, which is available to authorized users.

## Background

The growing population demands that plant biomass use becomes as efficient as possible, especially in large-scale applications: as a food and feed resource, and production of lignocellulosic biofuels and renewable materials [1–3]. Increasing the yield of sugars from both hemicelluloses and cellulose in the cell wall is important for the development of economic biorefineries and for use of improved plant biomass as an animal feed.

The intricate assembly and cross linking of lignin and polysaccharides within the cell wall renders the polysaccharides largely inaccessible to degradation [4]. Biomass digestion can be achieved by the use of expensive acid or alkali pretreatment, steam explosion or organic solvents [5], but these are not yet widely commercially viable processes. Consequently, there is considerable effort to increase the yield of sugar from biomass by breeding improved biomass crops, advancing pretreatment processes and improving enzyme cocktails. Despite substantial advances in all these areas, the lack of understanding of the molecular basis of recalcitrance prevents a targeted approach to improvement of saccharification. Cellulose is naturally resistant to enzymatic attack, but in the cell wall it is protected by hemicelluloses and lignin, which are removed in pretreatment processes to allow enzymatic saccharification of the cellulose [6]. Experiments using genetically modified plants and studies of genetic diversity have implicated lignin as one of the main cell wall components that influence digestibility [7–9].

In this work, we focussed on xylan, the major hemicellulose in secondary cell walls of eudicot angiosperms such as poplar, and an important hemicellulose in conifer cell walls, constituting up to 30 and 15% of dry material, respectively [10]. It is built of a β (1, 4)-linked xylose backbone that carries acetyl and [methyl]glucuronic acid ([Me]GlcA) branches in hardwoods and arabinose and MeGlcA substitutions in softwoods. In *Arabidopsis thaliana,* a model for hardwood secondary cell walls, xylan acetylation is believed to be catalysed by several acetyltransferases, of which TBL29/ESK is responsible for transfer of over 50% of acetyl groups [11]. The addition of α-1–2 linked GlcA branches to xylosyl residues is catalysed by GlucUronic acid substitution of Xylan (GUX) enzymes [12, 13]. The average degree of xylan glucuronosylation is 1 in every 8 xylosyl residues in angiosperms [14]. The frequency of [Me]GlcA in gymnosperms is higher, around 1 in every 6 xylose units [15]. GUX1/GUX2-deficient *Arabidopsis thaliana* plants (*gux1/2)* were shown to have no [Me]GlcA decorations on their secondary cell wall xylan [12]. GUX3 is responsible for glucuronosylation of xylan in primary walls [16]. GUX1, GUX2, and GUX4 enzymes show activity in vitro [13]. GlcA branches are 4-O-methylated by the activity of GXM enzymes [17, 18]. In our previous work, we demonstrated that extracted, deacetylated xylan from *gux1/2* plants can be completely digested by just two enzymes: a xylanase and a β-xylosidase [12]. This is due to simplification of this xylan substrate as xylosidases are inhibited by the presence of [Me]GlcA on wild-type xylan; complete digestion requires the additional action of α-glucuronidases [12, 19]. Commercial cocktails therefore contain xylan glucuronidases. Similarly, acetylation inhibits digestion of xylan, necessitating addition of acetyl esterases to cocktails [20].

Several attempts to modify xylan branching in vivo have had varying but limited success in improving cellulose and xylan digestibility. Reducing acetylation of xylan by about 50% caused strong dwarfing in the *tbl29*/*esk* mutant, and did not improve saccharification [11]. On the other hand, there was a small improvement in saccharification in the growth-suppressed *esk/kak* plants [21]. Similarly, partial in muro deacetylation of xylan with acetyl esterases in Arabidopsis showed modest increases in cellulose digestion [22]. Manipulation of GlcA methylation, by reducing it from 70 to 30% in the *gxm1* mutant, has also been reported to show a small increase in xylose release [17]. Attempts to remove [Me]GlcA in muro using a glucuronidase did not substantially change [Me]GlcA levels, or saccharification [23]. The *gux1/2* mutants show a small improvement in extractability of xylan with sodium hydroxide [12], but is not yet clear to what extent removal of [Me]GlcA branches could affect saccharification of cell walls.

We report here that both cellulose and xylan are much more easily digestible by the commercial enzymatic cocktail Cellic^®^ CTec2 in non-pretreated cell wall biomass of *gux1/2* plants than those of WT plants. Simultaneous saccharification and fermentation experiments demonstrated that sugars in the *gux1/2* biomass can be more efficiently converted to ethanol. To transfer this knowledge to industrially relevant plants, we identify and characterise a conifer GUX enzyme.

## Results

### Biomass lacking GlcA branches on xylan has reduced recalcitrance

**Fig. 1:**
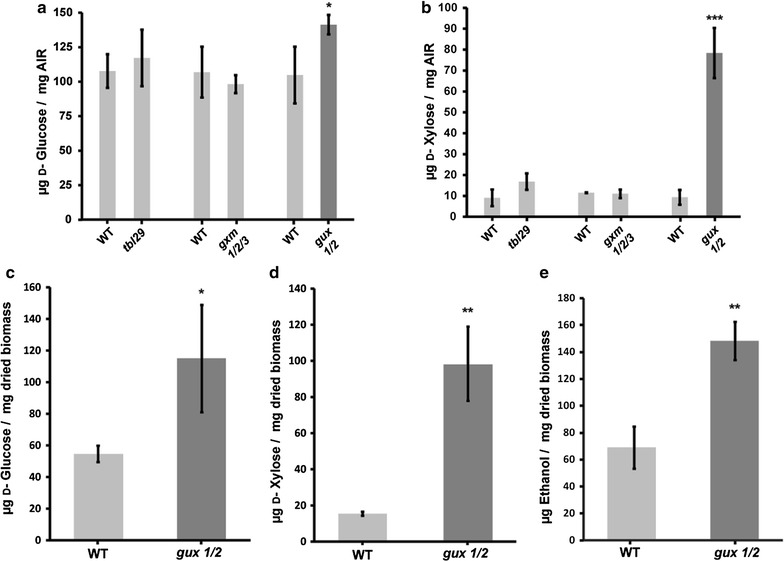
Biomass lacking xylan–[Me]GlcA decorations has reduced recalcitrance and is a superior feedstock for bioethanol production. Average d-glucose (**a**) and d-xylose (**b**) release following saccharification of WT, *tbl29, gxm1/2/3* and *gux1/2* AIR. d-glucose (**c**) and d-xylose release (**d**) from WT and *gux1/2* milled dried stems. Ethanol concentration after 96 h of simultaneous saccharification and fermentation of WT and *gux1/2* biomass (**e**). Ethanol yields were standardised for the readings from a fermentation reaction which did not include saccharification enzymes (Additional file 7: Table S1). Error bars represent standard deviation of three matching WT and mutant biological replicates of biomass, *p value ≤0.05; **p value ≤0.01; ***p value ≤0.001

To investigate the effect of modifying xylan decorations on recalcitrance, we performed saccharification experiments of modified stem biomass from *A. thaliana*. We studied plants with alterations in all the secondary cell wall xylan decorations: reduced acetylation (*tbl29/esk, 11),* without methylation of GlcA (*gxm1/2/3*, [24]) and without any [Me]GlcA sugar decorations (*gux1/2,* [12]). The reduced acetylation mutant is severely dwarfed [11], but the *gxm1/2/3* and *gux1/2* mutants grow without yield penalty (Additional file 1: Figure S1, Additional file 2: Figure S2). First, we measured sugar release from alcohol insoluble residues (AIR), a standard cell wall preparation involving milling in ethanol. Experiments used AIR without any further chemical pretreatment to avoid reducing any differences in biomass recalcitrance between plant genotypes. After enzymatic saccharification of AIR with the Cellic^®^ CTec2 enzyme cocktail, we observed increased release of both glucose (Fig. 1a) and xylose (Fig. 1b) from the *gux1/2* biomass in comparison with wild-type (WT). Remarkably, over 700% more xylose was released from *gux1/2* AIR (Fig. 1b). On the other hand, no statistically significant difference in sugar release was observed from either *tbl29* or *gxm1/2/3* plants in these saccharification conditions. Use of a feedstock without chemical pretreatment could reduce the environmental impact of biofuel production. Therefore, we next evaluated if the saccharification phenotype is also observed for wet-milled stems without any chemical pretreatments. After saccharification of WT and *gux1/2* milled stems, we observed double the release of glucose from the mutant biomass (Fig. 1c). Similarly to AIR saccharification, xylose release from *gux1/2* milled stems was improved five-fold (Fig. 1d). Thus, the [Me]GlcA decorations are critical for recalcitrance of biomass, and have an exceptionally large impact on xylose release.

A possible explanation for the *gux* saccharification phenotype is an increased amount of xylan in the stems of these plants. Some literature sources suggest an increase in xylose measurement for *gux1/2* by monosaccharide analysis [23]. We were unable to observe the same phenotype in our previous work [12]. However, this potential increase cannot fully explain the several-fold improvement in xylose yields observed in this work. Another possible explanation for increased xylose release from *gux1/2* might be insufficiency of glucuronidase activity in the enzyme mix used for saccharification. In this case, glucuronosylated oligosaccharides might accumulate in a reaction when WT feedstock is used, and such oligosaccharides would not be measured when xylose release is quantified. However, GH115 glucuronidase [19] treatment of Cellic^®^ CTec2 digested biomass, followed by second saccharification step, did not increase xylose release (Additional file 3: Figure S3). Therefore, the major improvement in xylan digestion in *gux1/2* biomass is likely to reflect alterations to the molecular architecture of the secondary cell walls.

To test if the improved monosaccharide yield can enhance bioethanol production, we carried out simultaneous saccharification and fermentation (SSF) experiments using wet-milled stems as a feedstock and transgenic *Escherichia coli* capable of ethanol production [25]. We used an *E. coli* strain that can utilise pentoses and produce ethanol. Thus, it is well suited to metabolise xylan saccharification products. Fermentation of simultaneously saccharified *gux1/2* biomass had double the yield of ethanol compared to ethanol from WT biomass SSF (Fig. 1e).

### Gymnosperm genomes encode putative GUX enzymes

**Fig. 2:**
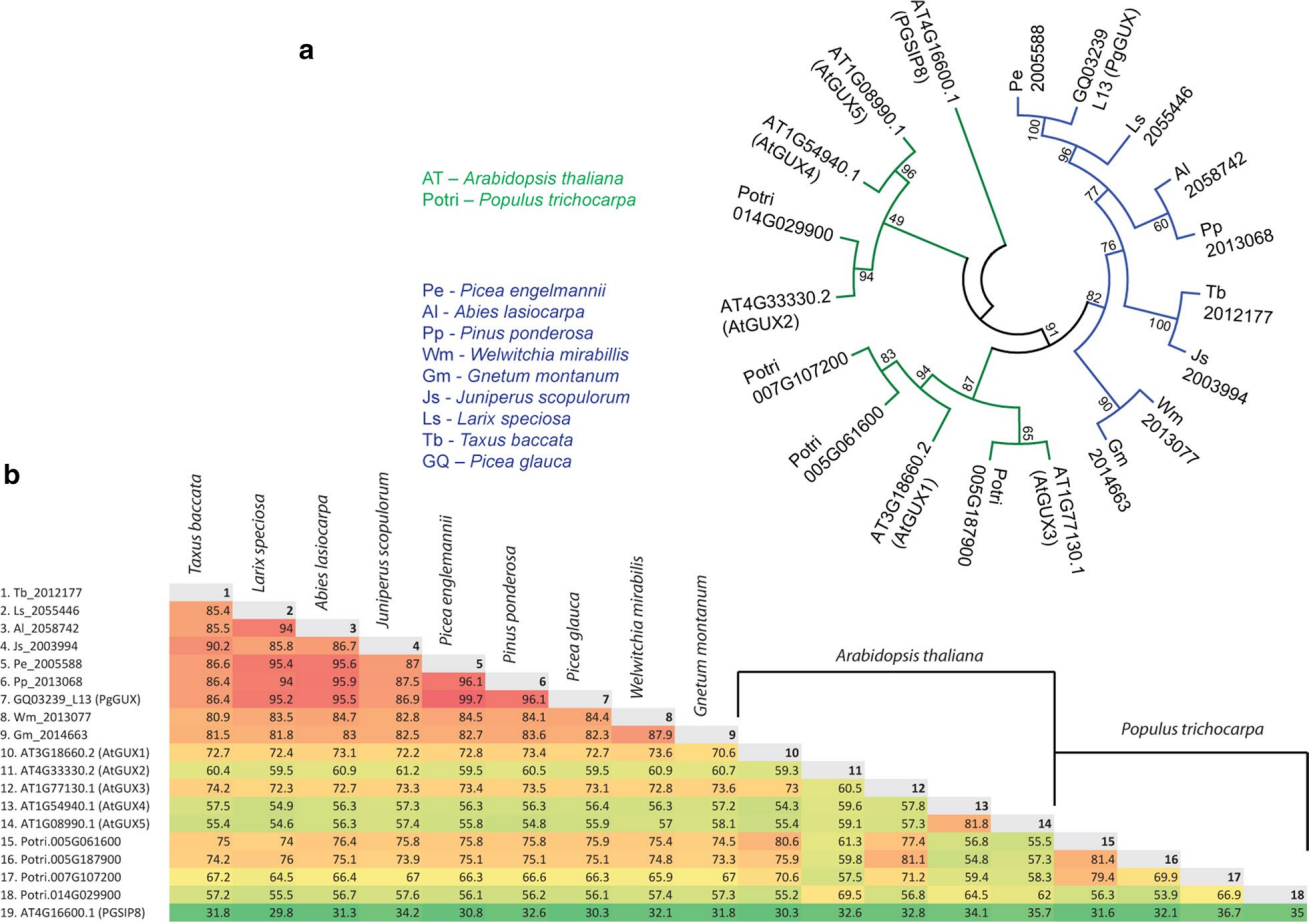
Identification of conifer GUX enzymes. **a** Phylogenetic analysis of putative dicot (green) and gymnosperm (blue) GUX enzymes. **b** Similarity matrix analysis for the gymnosperm and angiosperm GUX enzymes. A similarity score is indicated for each pair. Red colour denotes highest similarity. Pairs with lowest similarity are marked in green. PGSIP8, an* Arabidopsis* GT8 not in the GUX clade, was used as an outgroup

Xylan in hardwoods and softwoods contains patterned [Me]GlcA branches [15]. Conservation of this decoration suggests it plays an important function in all vascular plants [15]. Therefore, removing glucuronosylation from commercially relevant plants such as trees, including conifers that produce softwoods, could facilitate processing of wood into materials and biofuels. To identify possible conifer GUX glycosyltransferases, we examined sequences from the *P. glauca* transcriptome and found a read (PgGUX, clone: GQ03239_L13, GeneBank: BT111578.1) encoding a protein with up to 73% similarity to Arabidopsis GUX enzymes. Analysis of transcriptomic data from the OneKP project [26–29] identified reads encoding putative GUX enzymes of other gymnosperm species (Additional file 4: Table S2). A phylogenetic analysis of these newly identified gymnosperm putative GUX proteins showed that they are in a separate clade (Fig. 2a) and that they share a high degree of sequence similarity (Fig. 2b). Analysis of *Picea abies* transcriptome heat-map suggests that mRNA encoding a PgGUX homologue is enriched in wood supporting its function in xylan biosynthesis (Additional file 5: Figure S4) [30].

### PgGUX can decorate acetylated xylan in vitro

**Fig. 3:**
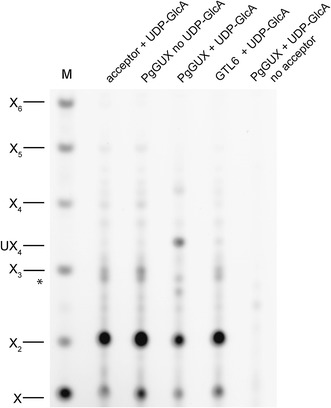
PgGUX has xylan glucuronosyltransferase activity in vitro. The assay was performed with UDP-GlcA, acetylated xylan without [Me]GlcA decorations, and microsomes from *N. benthamiana* expressing PgGUX or the control GTL6 protein. Products of the in vitro glucuronosylation were digested with xylanase GH11 and analysed by PACE. The enzyme generates xylose, xylobiose, plus the UX_4_ oligosaccharide if any GlcA is present on the xylan [12]. Average degree of glucuronisation was 10.9%. *Denote a background bands

To detect any PgGUX xylan glucuronosyltransferase activity, the enzyme was expressed as a *myc*-tagged protein in the tobacco *Nicotiana benthamiana* (Additional file 6: Figure S5). As a control for any endogenous tobacco glucuronosyltransferase activity, GTL6/MUCI10 (a Golgi-localised glucomannan galactosyltransferase, [31, 32]) was similarly expressed. Intact polymeric xylan from *gux1/2* lacking any [Me]GlcA decorations was used as an acceptor. Since this xylan is insoluble without acetylation, microsomes extracted from tobacco expressing PgGUX or the control GTL6 were incubated with acetylated *gux1/2* xylan and the reaction products were deacetylated and analysed by PACE using digestion with a GH11 xylanase. A GlcA-xylotetraose (UX_4_) product, indicating xylan glucuronosyltransferase activity, was observed for microsomes from PgGUX overexpressing plants incubated with the xylan acceptor in the presence of UDP-GlcA (Fig. 3).

### PgGUX is an active conifer xylan glucuronosyltransferase in vivo

**Fig. 4:**
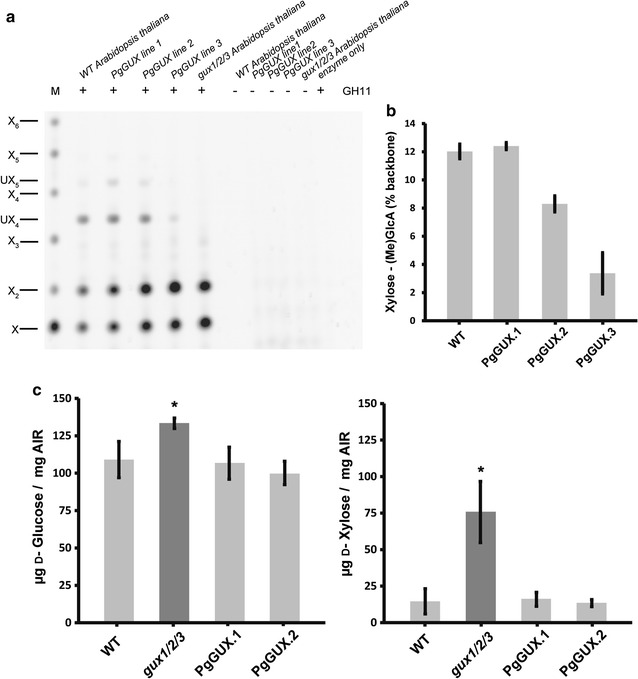
PgGUX is a functional xylan glucuronosyltransferase. **a** PACE analysis of GH11 xylanase digests of WT, three independent transgenic lines of PgGUX in *gux1/2/3* and control *gux1/2/3* AIR. Undigested AIR controls (−). The [Me]GlcA-xylotetraose band (UX_4_) was observed only in WT and PgGUX expressing lines. **b** Quantitation of degree of [Me]GlcA substitutions. **c** d-glucose and d-xylose release following saccharification of WT, *gux1/2/3* and two lines of PgGUX AIR. Error bars represent standard deviation of three biological replicates, *p value ≤0.05

Having established that PgGUX is active on acetylated xylan in vitro, we tested whether we could use it to introduce xylan decorations in vivo. The *PgGUX* coding sequence was placed under the control of the secondary cell wall specific Arabidopsis *IRX3* promoter. The construct was transformed into Arabidopsis *gux1/2/3* plants which lack [Me]GlcA branches on both primary and secondary cell wall xylan [16]. Use of these mutant plants ensured that any [Me]GlcA detected in the transgenic plants would be generated by the PgGUX enzyme. The degree of xylan glucuronosylation was evaluated by analysis of xylanase GH11 digestion products by PACE (Fig. 4a), as described [33]. In all three homozygous transgenic *gux1/2/3* lines expressing PgGUX, [Me]GlcA decorations were reintroduced (Fig. 4a, b). Thus, the cloned PgGUX enzyme is an active conifer glucuronosyltransferase in vivo.

To evaluate the recalcitrance of stem biomass from plants expressing PgGUX, we performed saccharification of AIR. In PgGUX lines, the monosaccharide release was reduced to levels measured for WT (Fig. 4c), indicating that the reintroduced glucuronosylation of the xylan is able to restore recalcitrance to the cell walls as effectively as native decorations.

## Discussion

Numerous studies have genetically modified plant cell walls to decrease recalcitrance but the improved saccharification has been moderate, or offset by yield penalties [34–36]. *Arabidopsis* without glucuronic acid decorations on xylan *(gux1/2*) shows no yield penalty (Additional file 2: Figure S2, [12, 16]). Here, we have demonstrated an exceptional increase in sugar release during enzymatic saccharification specifically for this xylan branching mutant. Moreover, there is a substantially improved SSF ethanol yield from chemically unpretreated *gux1/2* biomass.

In our work, we evaluated the impact of all modifications of dicot secondary cell wall xylan branching on biomass recalcitrance. No significant recalcitrance reduction was observed in plants with decreased xylan acetylation or methylation using our conditions, which is consistent with the no to moderate phenotypes previously reported with different saccharification conditions [11, 17]. We found that *gux1/2* is unique among xylan decoration mutants in yielding a strong improvement in sugar release following enzymatic saccharification. The effect is especially marked for xylan, where most of the xylan becomes enzymatically digestible without any chemical cell wall pretreatments. These observations strongly indicate *GUX* genes as prime candidates for designing biomass for biorefining. The molecular origins of biomass recalcitrance are not fully understood, but are thought to involve protection of the cellulose from enzyme attack through sheathing in hemicellulose and embedding in lignin [37–40]. The molecular dynamic simulations and acetylation pattern of xylan of the *gux1/2* mutant suggest that xylan is able to interact with cellulose microfibrils [41]. The unexpected sensitivity of recalcitrance to the presence of [Me]GlcA on xylan could be due to other factors, such as alteration of xylan linkages to lignin via a proposed [Me]GlcA ester [42, 43]. Alternatively, lack of [Me]GlcA on xylan may impact the relative positioning of cellulose microfibrils and lead to changes in lignin deposition patterns [44] which may result in reduction of biomass recalcitrance.

Softwood, an abundant biomass resource, has a simple pattern of [Me]GlcA decorations [15]. This suggests a possible lower complexity of the xylan biosynthesis machinery than in eudicots, which could be an advantage when engineering the conifer genome. We used a synthetic biology approach to characterise the xylan glucuronosyltransferase from *P. glauca* (PgGUX). PgGUX was able to glucuronosylate xylan in vitro and in the *gux1/2/3* mutant *Arabidopsis thaliana*. The efficiency of in vitro GlcA transfer by the PgGUX (Fig. 3) was comparable to degree of xylan glucuronisation *in planta* (Fig. 4b), showing unprecedented yield for a plant polysaccharide in vitro glycosylation reaction. Importantly, the [Me]GlcA decorations introduced by the PgGUX enzyme suppressed the improved saccharification phenotype observed in *gux1/2/3* plants, indicating that xylan glucuronosylation by PgGUX is functional.

Identification of a gymnosperm GUX enzyme is a major advance in the study of softwoods as a biomass resource and can contribute to the development of transgenic conifers more suited for biofuel production and biorefining. Not only the digestibility of cellulose can be improved in GUX-deficient Arabidopsis, but also their altered cell walls promote especially a more efficient utilisation of hemicelluloses, which form up to one-third of dry biomass. Additionally, many paper and pulp production processes remove hemicelluloses from wood using thermo-chemical treatment. It is likely that the efficiency of more environmentally benign pulping processes, such as enzymatic hemicellulose degradation, would be improved in GUX-deficient plants. Being a relatively simple alteration itself, *GUX* removal could be combined with other biomass modifications, further improving the quality of the lignocellulosic feedstock.

## Conclusion

As the improvements in saccharification and fermentation are especially prominent in *gux1/2* biomass without chemical treatment, our findings will promote innovation in more environmentally and economically sustainable processes of biomass use. Moreover, efficient saccharification without chemical pretreatment opens the possibility of using sugars from recalcitrant biomass such as wood as a feedstock for in vitro protein production, since toxic inhibitors are avoided with this pretreatment. As digestibility is improved in GUX-deficient plants, it may become possible to broaden the biomass that can contribute to animal feed, for example by using wood. With the growing population and pressure on land utilisation, such alternative uses of otherwise non-digestible biomass will become more significant. It is important to appreciate that different factors may determine recalcitrance of hardwoods and softwoods. Thus, softwood derived from conifers without functional copies of *PgGUX* homologues will need to be evaluated. Nonetheless, the discovery of [Me]GlcA importance for *A. thaliana* biomass recalcitrance together with identification of the softwood glucuronosyltransferase should provide a strong incentive for industrial and governmental organisations to invest in work aiming at engineering conifer genomes.

## Methods

### Plant material used and AIR preparation

*A*rabidopsis *thaliana* plants of the Columbia-0 ecotype were grown in a cabinet maintained at 21 °C, with a 16-h light, 8-h dark photoperiod. Mutant insertion lines described in [12] (*gux1/2)* and [16] (*gux1/2/3)* were used for saccharification and transformation experiments, respectively. Alcohol insoluble residue (AIR) was prepared from 5-cm-long sections of mature *A. thaliana* stem. All AIR preparation was carried out as described in [12].

### *A. thaliana* biomass saccharification using Cellic^®^ CTec2

Novozymes Cellic^®^ CTec2 (also available from Sigma-Aldrich/Merck) was used for all saccharification and fermentation experiments. Enzyme stock (35 µL) was diluted to a total volume of 2.5 mL with 0.1M ammonium acetate pH = 5.0 (AmAc) buffer. The enzyme sample was cleared from residual sugars using PD-10 desalting column (GE Healthcare) and eluted using 3.5 mL AmAc buffer, generating 1:00 (v/v) Cellic^®^ CTec2 solution. AIR aliquots (1 mg) were homogenised in 1 mL of AmAc buffer. Homogenised AIR was amended with 25 µL 1:100 Cellic^®^ CTec2 working solution.

For saccharification of dried stems 20 µL of 1:10 (v:v) Cellic^®^ CTec2 solution was used. The enzyme solution was added to 1 mg stem material/ml 0.1 M AmAc buffer. Stem suspension was generated by ball milling 8 mg of the biomass in 8 mL buffer for three periods of 10 min at 25 Hz, with 10-min intervals between each ball milling.

For both AIR and dried stems, saccharification was carried out for 24 h at 45 °C with 1400 rpm applied for 30 s every 4 min. The reaction was terminated by heat-treating the suspension at 100 °C for 10 min. d-Glucose and d-Xylose release from the biomass was quantified using commercial kits (Megazyme, catalogue codes: K-XYLOSE and K-GLUHK-220A). Sugar concentration for each experiment was standardised with readings obtained from biomass and enzyme only controls.

Glucuronidase supplementation was performed by incubating the products of Cellic^®^ CTec2 saccharification with 10 μL of 1 mg/mL *Bacteroides ovatus* GH115d (Bo_03449).

### Simultaneous saccharification and fermentation (SSF) experiments

50 mg dried stems of WT and *gux1/2* plants were used for each fermentation reaction. Stems were ball milled in 7 mL LB medium for 4 periods of 5 min at 20 Hz, with 5-min intervals between each ball milling cycle. The material was removed from ball milling vessel. To fully recover the biomass, the vessel was washed with further 2.5 mL LB. The stem suspension was sterilised by heat treatment at 85 °C for 10 min followed by cooling on ice. Each fermentation reaction was amended with 250 µL 1:10 Cellic^®^ CTec2 solution, prepared as described in saccharification section, and 250 µL of TOP10 *E. coli* inoculum bearing the BBa_K1122676 BioBrick (OD_600_ of the inoculum was within 0.55–0.6 range). BBa_K1122676 encodes a Pyruvate decarboxylase and Alcohol dehydrogenase from *Zymonomas mobilis* which allow ethanol production in *E.* *coli* [25]. Biomass only reactions were supplemented with 250 µL of AmAc buffer and the bacterial inoculum. The plasmid was maintained by provision of 25 µg/mL Chloramphenicol (Duchefa Biochemie). The simultaneous saccharification and fermentation reactions were carried out for 96 h. at 37 °C and 200 rpm. Fermentation vessel was kept air-tight throughout the experiment. Ethanol levels were analysed using a commercial kit (Megazyme, catalogue code: K-ETOH).

### Molecular phylogeny analysis of GUX amino acid sequences

The coding sequences of *A. thaliana GUX 1*, *2* and *3* were used to identify putative GUX encoding transcripts from *Populus trichocarpa* using data available via the NCBI BLAST service. The same *Arabidopsis* CDSs were used as a query to identify transcripts encoding putative GUX enzymes from Coniferophyta and Gnetophyta transcriptomic data available via 1000 Plant Genome BLAST service [26]. PgGUX transcript and amino acid sequences were retrieved from GeneBank. All amino acid sequences were reconstructed from transcripts with ExPASy translate tool and aligned using Multiple Sequence Comparison by Log-Expectation (MUSCLE) algorithm. A maximum likelihood phylogenetic tree was constructed using MEGA 6 software [45].

### Molecular cloning and generation of transgenic *A. thaliana* lines

The gene encoding the *P. glauca* enzyme with a 3xMyc C-terminal tag was synthesised by GeneScript. Gateway cloning was used to insert the gene into the p3KC binary vector [46]. Protein expression was driven by a 1.7 kbp promoter sequence of *A. thaliana IRX3* gene. *A. thaliana gux1/2/3* plants were transformed using the floral dip method [47]. Kanamycin resistant plants were screened for the construct using PCR (Forward primer: 5′-ACTCCCAGTTGGATCCTGTG-3′, Reverse primer: 5′-TCCATAAGCTGGAAGGT-3′). Three independent *gux1/2/3* lines homozygous for the pIRX3:PgGUX-Myc:NosT construct were derived and analysed in this study.

### Expression of PgGUX in *Nicothiana benthamiana*

PgGUX-3xMyc was amplified from the synthetic construct using Q5 DNA Polymerase (NEB, Forward primer: 5′-ATGAGGCCCTCTTCAGGAGTTC-3′, Reverse primer: 5′- TCAAAGCAAATCCTCTTCTGAGATCAGT-3′). PCR product was ligated into NruI (NEB) digested pEAQ-HT *N. benthamiana* overexpression vector [48] using T4 DNA ligase (Thermo-Fisher Scientific). The construct was transformed into competent AGL-1 *Agrobacterium tumefaciens* and infiltrated into *N. benthamiana* leaves according to a published protocol [49]. Leaves were harvested 3 days following the infiltration and the membranes fraction enriched for PgGUX was collected as described in [13]. Same protocol was followed for GTL6-3xMyc overexpression.

### Western blot analysis of PgGUX and GTL6 enriched membranes fraction

Protein concentration in the membranes fraction was quantified using modified Bradford reagent (Expedeon). Each well of SDS-PAGE (10–15% gradient, BioRad) was loaded with 2.5 or 5 μg of PgGUX or GTL6 enriched *N. benthamiana* leaf membrane protein. Following the run, the gel was transferred onto nitrocellulose membrane using iBlot system (Life Technologies). The membrane was blocked o/n in 5% milk in TBS solution. The following day it was probed with 1:2000 anti-Myc primary antibody (rabbit polyclonal, Santa-Cruz, A14) and with 1:10,000 mouse anti-rabbit HRP linked secondary antibody (Bio-Rad, 170-6515). Amersham ECL prime HRP substrate (GE-Lifesciences) was used to obtain signal from membrane bound antibodies.

### Glucuronosyltransferase activity assay

Acetylated heteroxylan lacking GlcA decorations was extracted from *gux1gux2 A. thaliana* as previously described [41]. Buffer exchange PD-10 columns (GE Lifesciences) were used to remove xylan from DMSO and elute it in water. Xylan aliquots were dried and used as an acceptor for in vitro GlcA transfer reaction. Each reaction mix was prepared as described in [16] with omission of UDP-Xylose. UDP-GlcA (5 mM) was replaced with water in certain reactions to control for non-specific glucuronosylation. Reaction was terminated with heat treatment (100 °C, 10 min) and the polysaccharides were extracted using methanol and chloroform as previously described [16]. Extracted polysaccharides were pelleted with 70% ethanol, washed in 100% ethanol and dried. Dry pellet was deacetylated with 4 M NaOH and digested with *Neocallimastix patriciarum* GH11 as previously described [12].

### Polysaccharide analysis by carbohydrate gel electrophoresis (PACE) of xylanase GH11 digestion products

AIR material (0.5 mg) was digested with *N. patriciarum* GH11 enzyme overnight as described in [12]. Released oligosaccharides were dried and derivatised with 8-aminonapthalene-1,3,6-trisulphonic acid (ANTS; Invitrogen). ANTS derivitasation, PACE running and visualisation were performed as previously described [50, 51].

### Statistical analysis and sampling

For the analysis of sugar release, fermentation efficiency and degree of GlcA substitution all plants were grown in three biological replicates. Each biological replicate consisted of a pooled sample of 36 plants. For each biological replicate, 3 technical replicates were analysed. Average sugar release/ethanol for each biological replicate was used for the statistical analysis with Student’s *T* test. The variance between each WT and mutant pair was estimated to be similar with Levene’s test.

## Additional files

**Additional file 1: Figure S1.** Analysis of *gxm 1/2/3* plants lacking methylation of GlcA. The triple mutant plants grow to the same height (**A**). The xylan (**B**) and GlcA (**C**) content of *gxm1/2/3* plants is not different when compared to WT. Growth images and xylan quantitation are representable for 3 biological replicates.

**Additional file 2: Figure S2.** Analysis of *gux1/2* plant growth and biomass production. Plant growth is not affected by removal of GlcA branches from xylan. The graphs represent average height of 7 week old plants (**A**, n = 36 for WT and 34 for *gux1/2*), average total plant mass (**B**, n = 36 for WT and 34 for *gux1/2*) and average mass of 5 cm basal stem sections (**C**, n = 36 for WT and 33 for *gux1/2*). Error bars represent standard deviation. There is no statistically significant difference between the values measured for the WT and the mutant plant (Student’s t test).

**Additional file 3: Figure S3.** Sugar release from WT and *gux1/2* biomass for saccharification reactions supplemented with glucuronidase GH115. Reaction scheme for GH115 supplementation (**A**) and D-Xylose release from Col0 (WT) and *gux1/2/3* biomass following supplementation (+) or not (-) with GH115 (**B**). Sterilisation steps were carried out between different stages of the experiment to avoid microbial growth in the reaction tubes. Reactions were performed in triplicate, and xylose measured after stage 1 or stage 3.

**Additional file 4: Table S2.** Gene Bank and OneKP transcript catalogue numbers. Those transcripts encode conifer GUX enzymes used to construct the maximum likelihood phylogeny presented in Fig. 2a.

**Additional file 5: Figure S4.** Expression heat-map for the putative conifer *GUX.* ExIMAGE feature of the Congenie datanase [30] was used to visualise the expression of *Picea abies* gene MA_84103g0010 which encodes a homologue of PgGUX. Reads encoding the enzyme are clearly enriched in both late and early wood.

**Additional file 6: Figure S5.** Western Blot analysis of *N. benthamiana* membrane fraction extracted from leaves enriched for GTL6 and PgGUX. PageRuler™ Prestained Protein Ladder, 10 to 180 kDa (Thermo-Fisher Scientific) was used as a molecular size marker.

**Additional file 7: Table S1.** Average ethanol production during Simultaneous saccharification and Fermentation experiments. This data was used to generate Fig. 1e.

## Abbreviations

AIR: alcohol insoluble residues
AmAc: ammonium acetate
ANTS: 8-aminonapthalene-1,3,6-trisulphonic acid
GH: glycosyl hydrolase
GlcA: glucuronic acid
GUX: glucuronic acid substitution of xylan
GXM: glucuronoxylan methyltransferase
HRP: horseradish peroxidase
IRX: irregular xylem
MeGlcA: 4-O-methylglucuronic acid
OneKP: one thousand plants
PACE: polysaccharide analysis by carbohydrate gel electrophoresis
Pg: *Picea glauca*
SSF: simultaneous saccharification and fermentation
TBL: trichome birefringence-like
UDP-Xyl: uridine[5′]diphospho-α-d-xylopyranoside
UX4: glucurono-xylotetraose
WT: wild-type

## References

[1] Albers SC, Berklund AM, Graff GD (2016). The rise and fall of innovation in biofuels. Nat Biotechnol.

[2] Cate HDJ, Ball AS (2016). Editorial overview: energy biotechnology. Curr Opin Biotechnol.

[3] Hood EE. Plant-based biofuels. F1000Res. 2016;17;5. pii: F1000 Faculty Rev-185.

[4] McCann MC, Carpita NC (2015). Biomass recalcitrance: a multi-scale, multi-factor, and conversion-specific property. J Exp Bot.

[5] Silveira MH, Morais AR, da Costa Lopes AM, Olekszyszen DN, Bogel-Łukasik R, Andreaus J, Pereira Ramos L (2015). Current pretreatment technologies for the development of cellulosic ethanol and biorefineries. Chem Sus Chem.

[6] Himmel ME (2008). Biomass recalcitrance: deconstructing the plant cell wall for bioenergy.

[7] Vanholme R, Morreel K, Darrah C, Oyarce P, Grabber JH, Ralph J, Boerjan W (2012). Metabolic engineering of novel lignin in biomass crops. New Phytol.

[8] Wang P, Dudareva N, Morgan JA, Chapple C (2015). Genetic manipulation of lignocellulosic biomass for bioenergy. Curr Opin Chem Biol.

[9] Mottiar Y, Vanholme R, Boerjan W, Ralph J, Mansfield SD (2016). Designer lignins: harnessing the plasticity of lignification. Curr Opin Biotechnol.

[10] Scheller HV, Ulvskov P (2010). Hemicelluloses. Annu Rev Plant Biol.

[11] Xiong G, Cheng K, Pauly M (2013). Xylan O-acetylation impacts xylem development and enzymatic recalcitrance as indicated by the *Arabidopsis* mutant tbl29. Mol Plant.

[12] Mortimer JC, Miles GP, Brown DM, Zhang Z, Segura MP, Weimar T, Yu X, Seffen KA, Stephens E, Turner SR, Dupree P (2010). Absence of branches from xylan in *Arabidopsis* gux mutants reveals potential for simplification of lignocellulosic biomass. PNAS.

[13] Rennie EA, Hansen SF, Baidoo EE, Hadi MZ, Keasling JD, Scheller HV (2012). Three members of the Arabidopsis glycosyltransferase family 8 are xylan glucuronosyltransferases. Plant Physiol.

[14] Brown DM, Goubet F, Wong VW, Goodacre R, Stephens E, Dupree P, Turner SR (2007). Comparison of five xylan synthesis mutants reveals new insight into the mechanisms of xylan synthesis. Plant J.

[15] Busse-Wicher M, Li A, Silveira RL, Pereira CS, Tryfona T, Gomes TC, Skaf MS, Dupree P (2016). Evolution of xylan substitution patterns in gymnosperms and angiosperms: implications for xylan interaction with cellulose. Plant Physiol.

[16] Mortimer JC, Faria-Blanc N, Yu X, Tryfona T, Sorieul M, Ng YZ, Zhang Z, Stott K, Anders N, Dupree P (2015). An unusual xylan in *Arabidopsis* primary cell walls is synthesised by GUX3, IRX9L, IRX10L and IRX14. Plant J.

[17] Urbanowicz BR, Peña MJ, Ratnaparkhe S, Avci U, Backe J, Steet HF, Foston M, Li H, O’Neill MA, Ragauskas AJ, Darvill AG, Wyman C, Gilbert HJ, York WS (2012). 4-O-methylation of glucuronic acid in Arabidopsis glucuronoxylan is catalyzed by a domain of unknown function family 579 protein. PNAS.

[18] Li X, Jackson P, Rubtsov DV, Faria-Blanc N, Mortimer JC, Turner SR, Krogh KB, Johansen KS, Dupree P (2013). Development and application of a high throughput carbohydrate profiling technique for analyzing plant cell wall polysaccharides and carbohydrate active enzymes. Biotechnol Biofuels..

[19] Rogowski A, Baslé A, Farinas CS, Solovyova A, Mortimer JC, Dupree P, Gilbert HJ, Bolam DN (2014). Evidence that GH115 α-glucuronidase activity, which is required to degrade plant biomass, is dependent on conformational flexibility. J Biol Chem.

[20] Zhang J, Siika-Aho M, Tenkanen M, Viikari L (2011). The role of acetyl xylan esterase in the solubilization of xylan and enzymatic hydrolysis of wheat straw and giant reed. Biotechnol Biofuels.

[21] Bensussan M, Lefebvre V, Ducamp A, Trouverie J, Gineau E, Fortabat MN, Guillebaux A, Baldy A, Naquin D, Herbette S, Lapierre C, Mouille G, Horlow C, Durand-Tardif M (2015). Suppression of dwarf and irregular xylem phenotypes generates low-acetylated biomass lines in *Arabidopsis*. Plant Physiol.

[22] Pawar PM, Derba-Maceluch M, Chong SL, Gómez LD, Miedes E, Banasiak A, Ratke C, Gaertner C, Mouille G, McQueen-Mason SJ, Molina A, Sellstedt A, Tenkanen M, Mellerowicz EJ (2016). Expression of fungal acetyl xylan esterase in *Arabidopsis thaliana* improves saccharification of stem lignocellulose. Plant Biotechnol J.

[23] Chong SL, Derba-Maceluch M, Koutaniemi S, Gómez LD, McQueen-Mason SJ, Tenkanen M, Mellerowicz EJ (2015). Active fungal GH115 α-glucuronidase produced in *Arabidopsis thaliana* affects only the UX1-reactive glucuronate decorations on native glucuronoxylans. BMC Biotechnol.

[24] Cornuault V, Buffetto F, Rydahl MG, Marcus SE, Torode TA, Xue J, Crépeau MJ, Faria-Blanc N, Willats WG, Dupree P, Ralet MC, Knox JP (2015). Monoclonal antibodies indicate low-abundance links between heteroxylan and other glycans of plant cell walls. Planta.

[25] Lewicka AJ, Lyczakowski JJ, Blackhurst G, Pashkuleva C, Rothschild-Mancinelli K, Tautvaišas D, Thornton H, Villanueva H, Xiao W, Slikas J, Horsfall L, Elfick A, French C (2014). Fusion of pyruvate decarboxylase and alcohol dehydrogenase increases ethanol production in *Escherichia coli*. ACS Synth Biol..

[26] Wickett NJ (2014). Phylotranscriptomic analysis of the origin and early diversification of land plants. PNAS.

[27] Matasci N (2014). Data access for the 1000 plants (1KP) project. GigaScience.

[28] Xie Y (2014). SOAPdenovo-Trans: de novo transcriptome assembly with short RNA-Seq reads. Bioinformatics.

[29] Johnson MT (2012). Evaluating methods for isolating total RNA and predicting the success of sequencing phylogenetically diverse plant transcriptomes. PLoS ONE.

[30] Nystedt B (2013). The Norway spruce genome sequence and conifer genome evolution. Nature.

[31] Dunkley TP, Hester S, Shadforth IP, Runions J, Weimar T, Hanton SL, Griffin JL, Bessant C, Brandizzi F, Hawes C, Watson RB, Dupree P, Lilley KS (2006). Mapping the *Arabidopsis organelle* proteome. PNAS.

[32] Voiniciuc C, Schmidt MH, Berger A, Yang B, Ebert B, Scheller HV, North HM, Usadel B, Günl M (2015). Mucilage-Related10 produces galactoglucomannan that maintains pectin and cellulose architecture in arabidopsis seed mucilage. Plant Physiol.

[33] Brown DM, Zhang Z, Stephens E, Dupree P, Turner SR (2009). Characterization of IRX10 and IRX10-like reveals an essential role in glucuronoxylan biosynthesis in *Arabidopsis*. Plant J.

[34] Wang Y, Fan C, Hu H, Li Y, Sun D, Wang Y, Peng L (2016). Genetic modification of plant cell walls to enhance biomass yield and biofuel production in bioenergy crops. Biotechnol Adv.

[35] Marriott PE, Gómez LD, McQueen-Mason SJ (2016). Unlocking the potential of lignocellulosic biomass through plant science. New Phytol.

[36] Loqué D, Scheller HV, Pauly M (2015). Engineering of plant cell walls for enhanced biofuel production. Curr Opin Plant Biol.

[37] Öhgren K, Bura R, Saddler J, Zacchi G (2007). Effect of hemicellulose and lignin removal on enzymatic hydrolysis of steam pretreated corn stover. Biores Technol.

[38] Fernandes AN, Thomas LH, Altaner CM, Callow P, Forsyth VT, Apperley DC, Kennedy CJ, Jarvis MC (2011). Nanostructure of cellulose microfibrils in spruce wood. PNAS.

[39] Cosgrove DJ, Jarvis MC (2012). Comparative structure and biomechanics of plant primary and secondary cell walls. Front Plant Sci.

[40] Simmons TJ, Mortimer JC, Bernardinelli OD, Pöppler AC, Brown SP, deAzevedo ER, Dupree R, Dupree P (2016). Folding of xylan onto cellulose fibrils in plant cell walls revealed by solid-state NMR. Nat Commun.

[41] Busse-Wicher M, Gomes TC, Tryfona T, Nikolovski N, Stott K, Grantham NJ, Bolam DN, Skaf MS, Dupree P (2014). The pattern of xylan acetylation suggests xylan may interact with cellulose microfibrils as a twofold helical screw in the secondary plant cell wall of *Arabidopsis thaliana*. Plant J.

[42] Giummarella N, Lawoko M (2016). Structural basis for the formation and regulation of lignin-xylan bonds in birch. ACS Sustain Chem Eng.

[43] Watanabe T, Koshijima T (1988). Evidence for an ester linkage between lignin and glucuronic-acid in lignin carbohydrate complexes by DDQ-oxidation. Agric Biol Chem.

[44] Reis D, Thellier M (2004). Helicoidal pattern in secondary cell walls and possible role of xylans in their construction. C R Biol.

[45] Tamura K, Stecher G, Peterson D, Filipski A, Kumar S (2013). MEGA6: molecular evolutionary genetics analysis version 6.0. Mol Biol Evol.

[46] Atanassov II, Atanassov II, Etchells JP, Turner SR (2009). A simple, flexible and efficient PCR-fusion/Gateway cloning procedure for gene fusion, site-directed mutagenesis, short sequence insertion and domain deletions and swaps. Plant Methods.

[47] Clough SJ, Bent AF (1998). Floral dip: a simplified method for Agrobacterium-mediated transformation of *Arabidopsis thaliana*. Plant J.

[48] Sainsbury F, Thuenemann EC, Lomonossoff GP (2009). pEAQ: versatile expression vectors for easy and quick transient expression of heterologous proteins in plants. Plant Biotechnol J.

[49] Sparkes IA, Runions J, Kearns A, Hawes C (2006). Rapid, transient expression of fluorescent fusion proteins in tobacco plants and generation of stably transformed plants. Nat Protoc.

[50] Bromley JR, Busse-Wicher M, Tryfona T, Mortimer JC, Zhang Z, Brown DM, Dupree P (2013). GUX1 and GUX2 glucuronyltransferases decorate distinct domains of glucuronoxylan with different substitution patterns. Plant J.

[51] Goubet F, Jackson P, Deery MJ, Dupree P (2002). Polysaccharide analysis using carbohydrate gel electrophoresis: a method to study plant cell wall polysaccharides and polysaccharide hydrolases. Anal Biochem.

