# Supplementary figures and images for: Removal of glucuronic acid from xylan is a strategy to improve the conversion of plant biomass to sugars for bioenergy
Source: Biotechnol Biofuels. 2017 Sep 19;10:224. doi: 10.1186/s13068-017-0902-1 (PMC5606085; doi:10.1186/s13068-017-0902-1)

A)

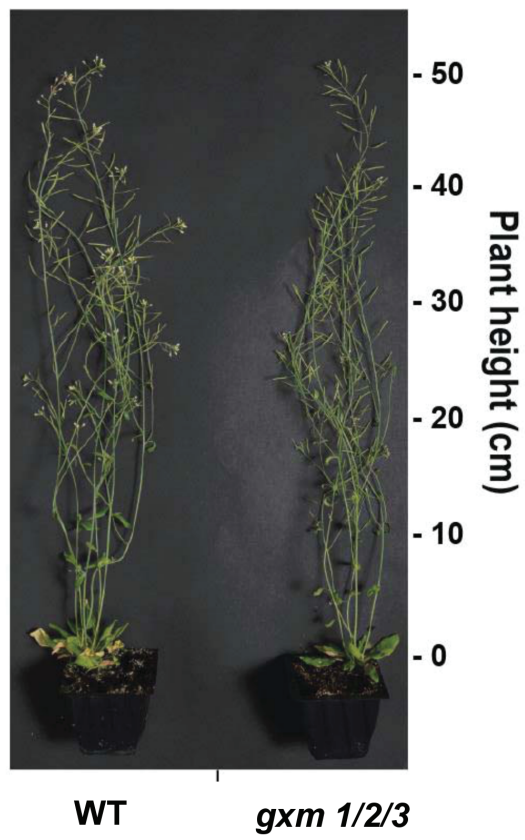

B)

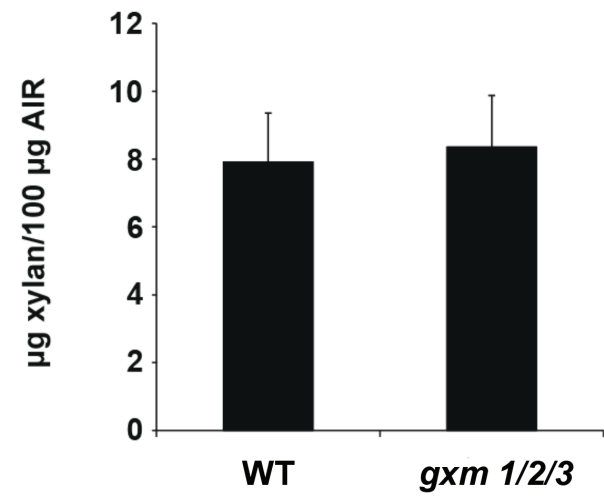

C)

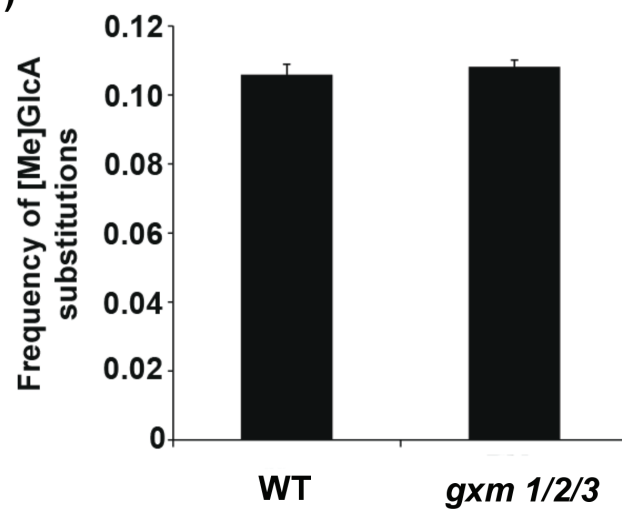

Supplement: Supplementary file 1 — Additional file 1: Figure S1. Analysis of gxm 1/2/3 plants lacking methylation of GlcA. The triple mutant plants grow to the same height (A). The xylan (B) and GlcA (C) content of gxm1/2/3 plants is not different when compared to WT. Growth images and xylan quantitation are representable for 3 biological replicates. [file 13068_2017_902_MOESM1_ESM.pdf]

A)

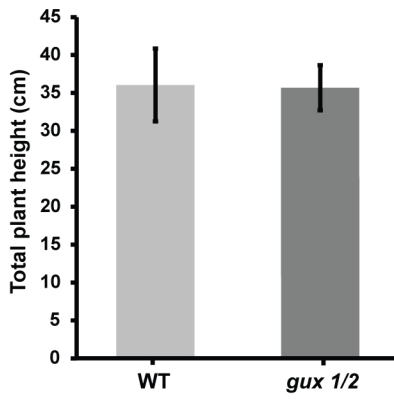

B)

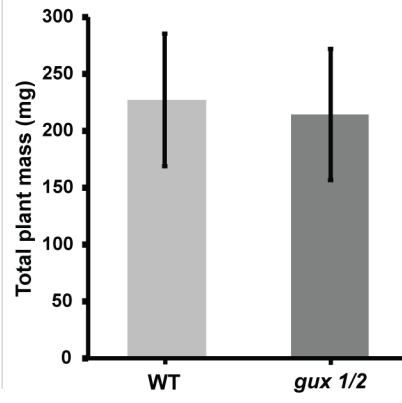

C)

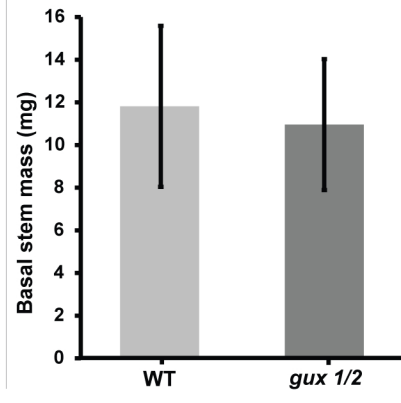

Supplement: Supplementary file 2 — Additional file 2: Figure S2. Analysis of gux1/2 plant growth and biomass production. Plant growth is not affected by removal of GlcA branches from xylan. The graphs represent average height of 7 week old plants (A, n = 36 for WT and 34 for gux1/2), average total plant mass (B, n = 36 for WT and 34 for gux1/2) and average mass of 5 cm basal stem sections (C, n = 36 for WT and 33 for gux1/2). Error bars represent standard deviation. There is no statistically significant difference between the values measured for the WT and the mutant plant (Student’s t test). [file 13068_2017_902_MOESM2_ESM.pdf]

A)

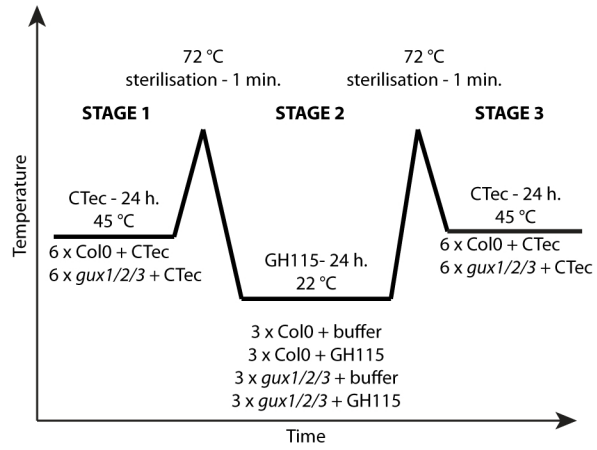

B)

### D-Xylose - C Tec GH115 supplementation

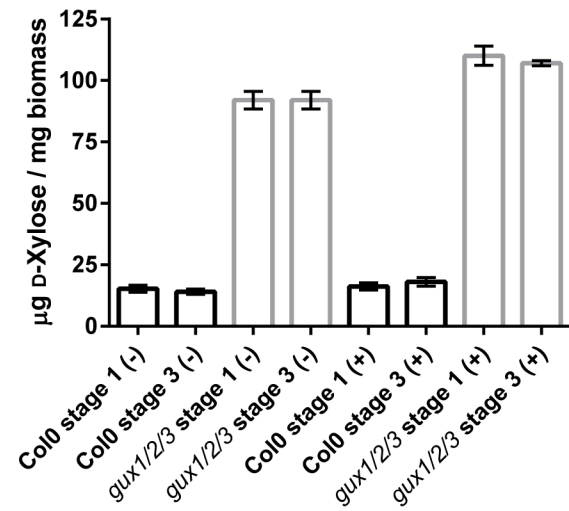

Samples +/- GH115 treatment during Stage 2

Supplement: Supplementary file 3 — Additional file 3: Figure S3. Sugar release from WT and gux1/2 biomass for saccharification reactions supplemented with glucuronidase GH115. Reaction scheme for GH115 supplementation (A) and D-Xylose release from Col0 (WT) and gux1/2/3 biomass following supplementation (+) or not (-) with GH115 (B). Sterilisation steps were carried out between different stages of the experiment to avoid microbial growth in the reaction tubes. Reactions were performed in triplicate, and xylose measured after stage 1 or stage 3. [file 13068_2017_902_MOESM3_ESM.pdf]

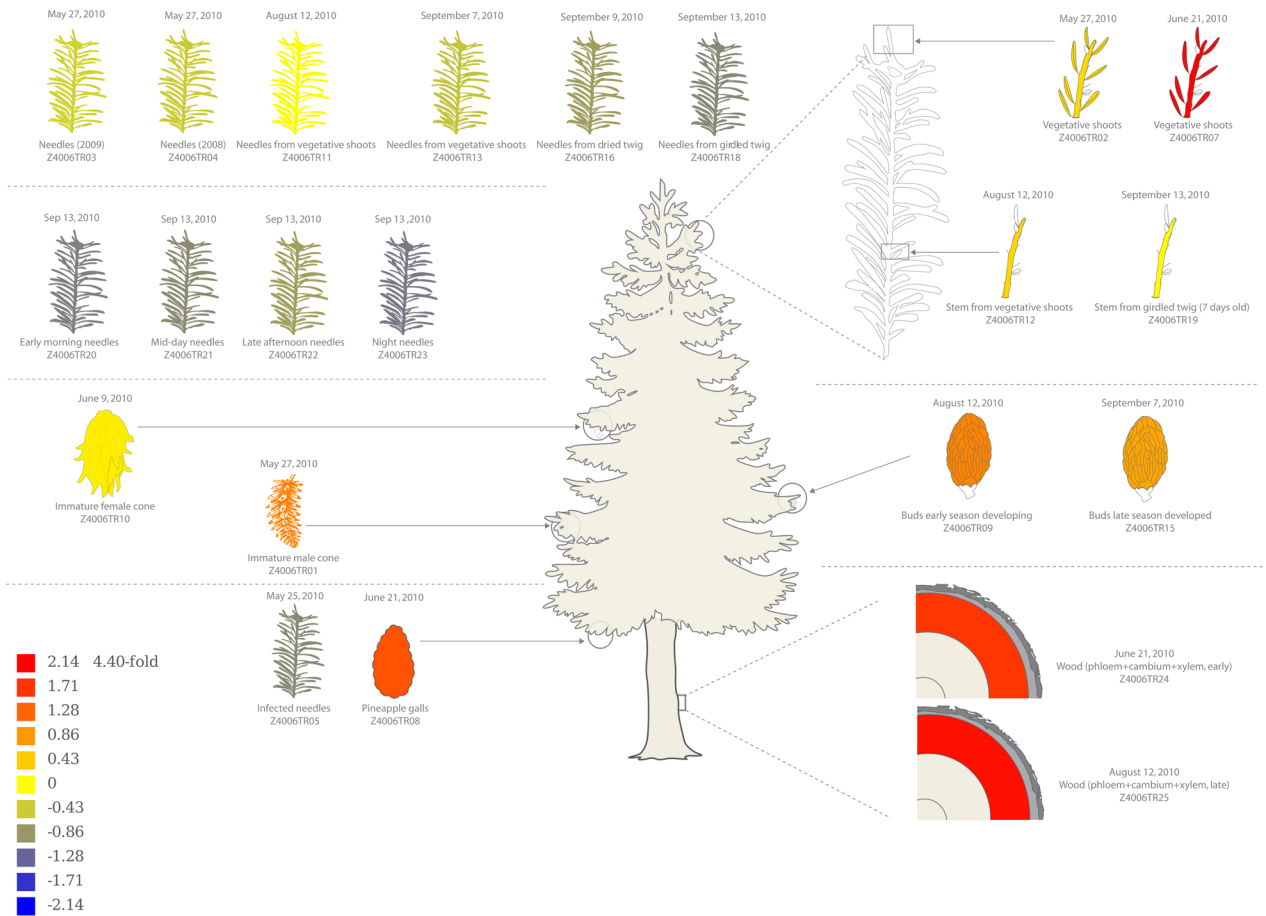

Supplement: Supplementary file 5 — Additional file 5: Figure S4. Expression heat-map for the putative conifer GUX. ExIMAGE feature of the Congenie datanase [30] was used to visualise the expression of Picea abies gene MA_84103g0010 which encodes a homologue of PgGUX. Reads encoding the enzyme are clearly enriched in both late and early wood. [file 13068_2017_902_MOESM5_ESM.pdf]

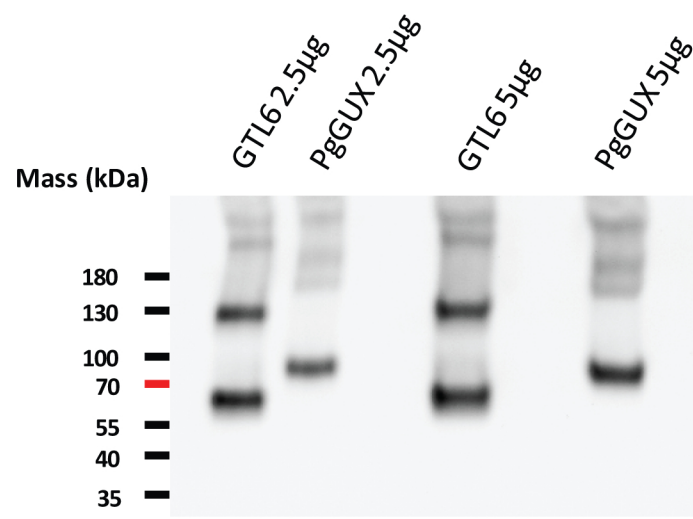

Supplement: Supplementary file 6 — Additional file 6: Figure S5. Western Blot analysis of N. benthamiana membrane fraction extracted from leaves enriched for GTL6 and PgGUX. PageRuler™ Prestained Protein Ladder, 10 to 180 kDa (Thermo-Fisher Scientific) was used as a molecular size marker. [file 13068_2017_902_MOESM6_ESM.pdf]
