# Supplementary material for: Removal of glucuronic acid from xylan is a strategy to improve the conversion of plant biomass to sugars for bioenergy
Source: Biotechnol Biofuels. 2017 Sep 19;10:224. doi: 10.1186/s13068-017-0902-1 (PMC5606085; doi:10.1186/s13068-017-0902-1)
Supplement: Supplementary file 4 — Additional file 4: Table S2. Gene Bank and OneKP transcript catalogue numbers. Those transcripts encode conifer GUX enzymes used to construct the maximum likelihood phylogeny presented in Fig. 2a. [file 13068_2017_902_MOESM4_ESM.pdf]

**Putative GUX enzyme code**	**GeneBank/ OneKP code**
Abies lasiocarpa 2058742	VSRH-2058742 (OneKP)
Gnetum montanum 2014663	GTHK-2014663 (OneKP)
Juniperus scopulorum 2003994	XMGP-2003994 (OneKP)
Larix speciosa 2055446	WVWN-2055446 (OneKP)
PgGUX	GQ03239 (GeneBank)
Picea englemanii 2005588	AWQB-2005588 (OneKP)
Pinus ponderosa 2013068	JBND-2013068 (OneKP)
Taxus baccata 2012177	WWSS-2012177 (OneKP)
Welwitchia mirabillis 2013077	TOXE-2013077 (OneKP)
