# Supplementary material for: Removal of glucuronic acid from xylan is a strategy to improve the conversion of plant biomass to sugars for bioenergy
Source: Biotechnol Biofuels. 2017 Sep 19;10:224. doi: 10.1186/s13068-017-0902-1 (PMC5606085; doi:10.1186/s13068-017-0902-1)
Supplement: Supplementary file 7 — Additional file 7: Table S1. Average ethanol production during Simultaneous saccharification and Fermentation experiments. This data was used to generate Fig. 1e. [file 13068_2017_902_MOESM7_ESM.pdf]

Sample	Average Ethanol production (µg/ mg biomass, standard deviation)
Cellic ® CTec2 only	151 (±11)
WT no Cellic ® CTec2	154 (±23)
gux1/2 no Cellic ® CTec2	157 (±20)
WT with Cellic ® CTec2	223 (±24)
gux1/2 with Cellic ® CTec2	305 (±28)
